# ?Holothuria (Lessonothuria) coronata sp. nov. (Echinodermata, Holothuroidea), a new species of sea cucumber from Socorro Island, México

**DOI:** 10.3897/zookeys.1095.79375

**Published:** 2022-04-13

**Authors:** Brenda Anaid Yáñez Villanueva, Francisco Alonso Solís-Marín, Alfredo Laguarda-Figueras

**Affiliations:** 1 Posgrado en Ciencias Biológicas, Universidad Nacional Autónoma de México, Mexico City, Mexico Universidad Nacional Autónoma de México Mexico City Mexico; 2 Colección Nacional de Equinodermos ‘Dra. Ma. Elena Caso Muñoz’, Laboratorio de Sistemática y Ecología de Equinodermos, Instituto de Ciencias del Mar y Limnología (ICML), Universidad Nacional Autónoma de México (UNAM), Mexico City, C.P. 04510, Mexico Universidad Nacional Autónoma de México Mexico City Mexico

**Keywords:** Holothuriidae, Revillagigedo Archipelago, morphology, ossicles, taxonomy, Archipiélago Revillagigedo, espículas, Holothuriidae, morfología, Taxonomía

## Abstract

Holothuria (Lessonothuria) coronata**sp. nov.** occurs in depths of 5–10 m off the Mexican Pacific coast at the Revillagigedo Archipelago. It is clearly distinguished from other species of the subgenus by the presence of tables with a circular disc and big peripheral holes, sometimes with a second series of peripheral ones, a disc with a spiny or smooth rim and a spire crossed by a single cross-beam, dorsal papillae, and ventral tube feet with curved supporting rods with a spiny edge.

## Abstract (fr)

Holothuria (Lessonothuria) coronata**sp. nov.** habita a profundidades de 5–10 m frente a la costa del Pacífico mexicano en el archipiélago de Revillagigedo. Se distingue claramente de otras especies del subgénero por la presencia de tablas con disco circular y grandes orificios periféricos, a veces con una segunda serie de orificios periféricos, disco con borde espinoso o liso y espira con una sola viga transversal, papilas dorsales y pies ambulacrales ventrales con barrotes de soporte curvados, con borde espinoso.

## ?Introduction

The genus *Holothuria* Linnaeus, 1767 is the most diverse within the family Holothuriidae, with 163 of the 202 species in the family belonging to the genus (WoRMS 2021a, b, c, d, e). Currently, 18 subgenera are grouped in the genus *Holothuria* (WoRMS 2021c). In 1958, Deichmann erected the genus *Lessonothuria* and assigned *Holothuriapardalis* Selenka, 1867 as the type species. Contrary to Deichmann (1958), Rowe (1969) changed the category of *Lessonothuria* from genus to subgenus.

The ossicles of the species belonging to *Lessonothuria* consist of tables, regular to incomplete buttons and supporting rods that sometimes are modified into elongated buttons (Deichmann 1958). According to Rowe (1969), pseudo-buttons are abundant among the species of this subgenus. Species of this subgenus mostly inhabit shallow marine waters (Samyn 2003; Ahmed et al. 2020); nonetheless, H. (L.) cavans Massin & Tomascik, 1996 inhabits waters at low salinities in an anchialine lagoon (Massin and Tomascik 1996). Species grouped in this subgenus are mostly distributed in the Indian Ocean (Samyn et al. 2019; Ahmed et al. 2020).

Samyn et al. (2019) resurrected the species H. (L.) lineata Ludwig, 1875, which was considered as a synonym of *H.pardalis* Selenka, 1867. The authors also stated that pseudo-buttons are predominant in *H.insignis* Ludwig, 1875, while buttons are predominant in *H.lineata* Ludwig, 1875 and *H.pardalis* Selenka, 1867. Recently, Ahmed et al. (2020) formally resurrected *H.insignis* Ludwig, 1875, which was considered a synonym of *H.lineata* Ludwig, 1875 and *H.pardalis* Selenka, 1867.

Currently, only 11 species belong to the subgenus Lessonothuria Deichmann, 1958: Holothuria (L.) cavans Massin & Tomascik, 1996; H. (L.) cumulus Clark, 1921; H. (L.) duoturricula Cherbonnier, 1988; H. (L.) glandifera Cherbonnier, 1955; H. (L.) immobilis Semper, 1868; H. (L.) insignis Ludwig, 1875; H. (L.) lineata Ludwig, 1875; H. (L.) multipilula Liao, 1975; H. (L.) pardalis Selenka, 1867; H. (L.) tuberculata Thandar, 2007 and H. (L.) verrucosa Selenka, 1867 (WoRMS 2021c). However, Ahmed et al. (2020) concluded that some of these species probably do not belong to this subgenus.

The purpose of this paper is to describe a new species of Holothuria (Lessonothuria) from the eastern Pacific coast.

## ?Materials and methods

Specimens were collected by snorkelling, relaxed in ~10% MgCl_2_ solution, and preserved in 70% ethanol for morphological and ossicle examination. All measurements were obtained from fixed specimens. Ossicles were extracted from the body wall (anterior, medium, and posterior regions), dorsal papillae, ventral tube feet, and tentacles. The tissue was dissolved in fresh household bleach (5–6.5%). After centrifugation at 1000 rpm for 10 min, bleach was pipetted off and the ossicles were rinsed and centrifuged with distilled water that was pipetted off afterwards. The same process was done with 70, 80, and 95% ethanol. Absolute ethanol was added to the ossicles, and a small aliquot was taken and placed to dry on a cylindrical double-coated conductive carbon tape stub. Then, it was sputter coated with gold 2.5 kV in the ionizer JEOL JFC-1100 for 3 min and photographed using a JEOL JSM-6360LV scanning electron microscope (SEM) at the ICML, UNAM. Specimens were deposited at the Colección Nacional de Equinodermos ‘Dra. Ma. Elena Caso Muñoz’, Instituto de Ciencias del Mar y Limnología, Universidad Nacional Autónoma de México, Mexico City (**ICML-UNAM**).

## ?Taxonomy

### ?Order Holothuriida Miller et al., 2017

**Family Holothuriidae Burmeister, 1837**

#### Genus *Holothuria* Linnaeus, 1767

##### Subgenus Lessonothuria

Taxon classificationAnimaliaHolothuriidaHolothuriidae

Deichmann, 1958

EBF967E4-7800-5F61-9237-E1C512DA8205

###### Diagnosis

**(after Rowe 1969).** Tentacles 17–30. Pedicels and papillae irregularly arranged ventrally and dorsally respectively, a ‘collar’ of papillae evident around the base of the tentacles, anal papillae usually apparent. Body wall soft, not very thick, usually 1 (1–3) mm, body almost cylindrical but with a more or less distinct, flattened sole. Size small to moderate, up to 150 mm long. Calcareous ring fairly stout, radial plates about twice as long as the interradial plates. Ossicles consisting of clumsy tables, the spire low to moderate and usually terminating in a ring or cluster of spines, disc well developed and spinose, rarely some tables with smooth-rimmed disc also present; rim often turned up to give a ‘cup and saucer’ appearance to the table in lateral view. Pseudo-buttons abundant, usually smooth, sometimes spinose, usually irregular in outline and often reduced to a single row of three or four holes. Occasionally, quite regular buttons are present, with three pairs of holes.

###### Type species.

*Holothuriapardalis* Selenka, 1867 (original designation).

##### ? Holothuria (Lessonothuria) coronata sp. nov.

Taxon classificationAnimaliaHolothuriidaHolothuriidae

6A75825C-1F4E-543E-AD83-31268388DCF5

http://zoobank.org/0734862E-3F19-4790-AD3B-04440C36B869

Figs 1 , 2 , 3 , 4

###### Type material.

***Holotype***ICML-UNAM 18432, 49 mm total length (TL), 1 specimen, Bahía Vargas Lozano, Isla Socorro, Revillagigedo Archipelago, Pacific Ocean 18°43'89.3"N, 110°57'30.3"W, 1 m depth, 4 April 2014, coll. Estrada Galicia, D. & Isaac Novoa, Y. ***Paratypes*.**ICML-UNAM 18433, 48 mm TL, Bahía Vargas Lozano, Isla Socorro, Revillagigedo Archipelago, Pacific Ocean 18°43'89.3"N, 110°57'30.3"W, 0.5–8 m depth, 4 April 2014; coll. Estrada Galicia, D. & Isaac Novoa, Y.; ICML-UNAM 18434, 48 mm TL; 1 specimen, Bahía Vargas Lozano, Isla Socorro, Revillagigedo Archipelago, Pacific Ocean 18°43'29.14"N, 110°56'57.77"W, 5–10 m depth, 29 May 2010, coll. Estrada Galicia, D. & Isaac Novoa, Y.

**Figure 1.**
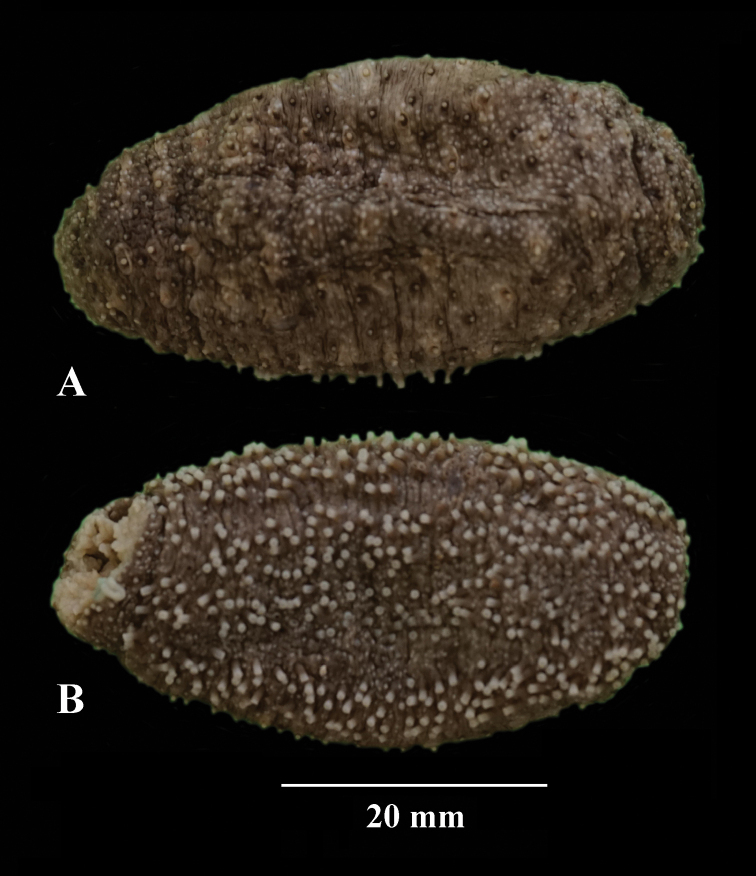
Holothuria (Lessonothuria) coronata sp. nov. Photos of preserved holotype, ICML-UNAM 18432 **A** dorsal view **B** ventral view.

###### Type locality.

Bahía Vargas Lozano, Isla Socorro, Revillagigedo Archipelago, Pacific Ocean 18°43'29.14"N, 110°56'57"W.

###### Diagnosis.

Small-sized holothurian (up to 49 mm), 20 peltate tentacles, no distinct collar; anal papillae present. Podia as conical papillae dorsally and tube feet ventrally. Body wall thin, leathery. Single Polian vesicle, free stone canal. Ossicles as tables, buttons, pseudo-buttons and supporting rods, tube feet with perforated plates. Table discs spinose to smooth with 6–13 marginal holes; spire of moderate height, one cross-beam, terminating in a crown with 14–20 teeth, such crown with a high shape diversity. Pseudo-buttons (25–50 µm long) mostly irregular, often twisted and/or knobbed, with two to five holes. Dorsal papillae and tube feet with curved supporting rods (180–240 µm), ends and central part are widened and perforated, the edge of the widened part slightly spinose. Tentacles with slightly curved rods, spiny ends, without perforations (300–600 µm).

###### Holotype description.

Specimen preserved in alcohol with brownish body wall, the papillae, and ventral tube feet are white; 20 short beige tentacles. Ventral and dorsal body wall with minute white dots due to the presence of ossicle clusters. Anus surrounded by anal papillae. Calcareous ring stout (Fig. 4), interradial pieces with a median anterior projection; radial pieces with a median anterior notch and much wider than the interradial pieces; dorsal radial pieces are wider and stouter than the ventral ones. Polian vesicle 10 mm long. Free stone canal, 2 mm long. Single madreporite. Longitudinal muscles divided, 8–10 mm wide. Tentacular ampullae 5 mm long. Cuvierian organs present.

**Figure 2.**
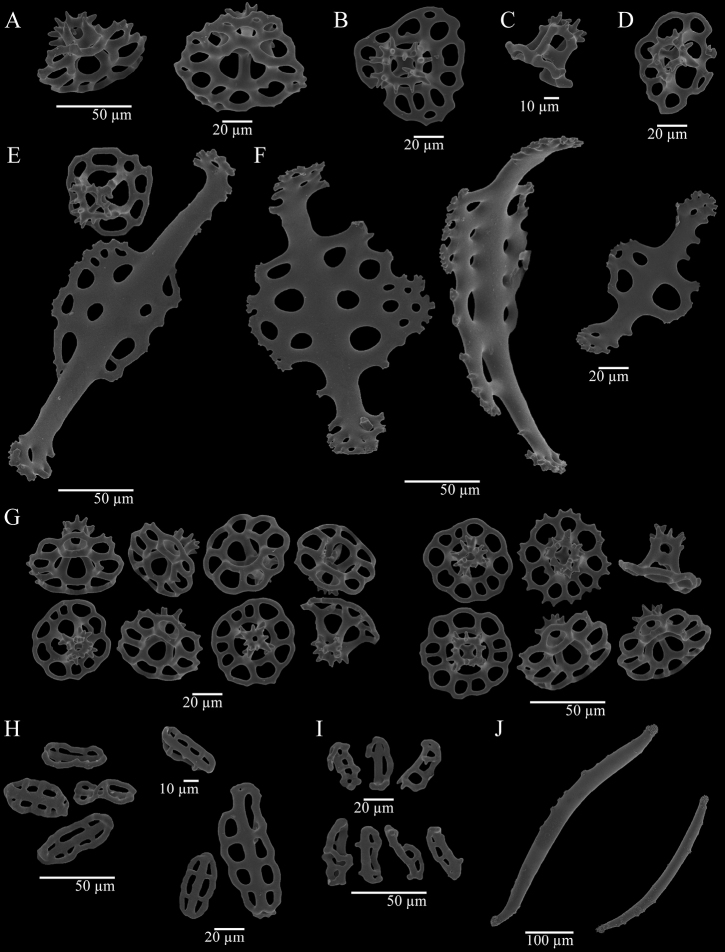
Holothuria (Lessonothuria) coronata sp. nov. Holotype, ICML-UNAM 18432. Ossicles of dorsal papillae (**A–F**) **A** tables **B** table with irregular disc **C** table with reduced disc **D** ‘rare table’ of the dorsal **E** supporting rod and table **F** supporting rods. Ossicles of dorsal body wall (**G–I**) **G** tables **H** pseudo-buttons **I** buttons. Ossicles of tentacles **J** rods.

**Figure 3.**
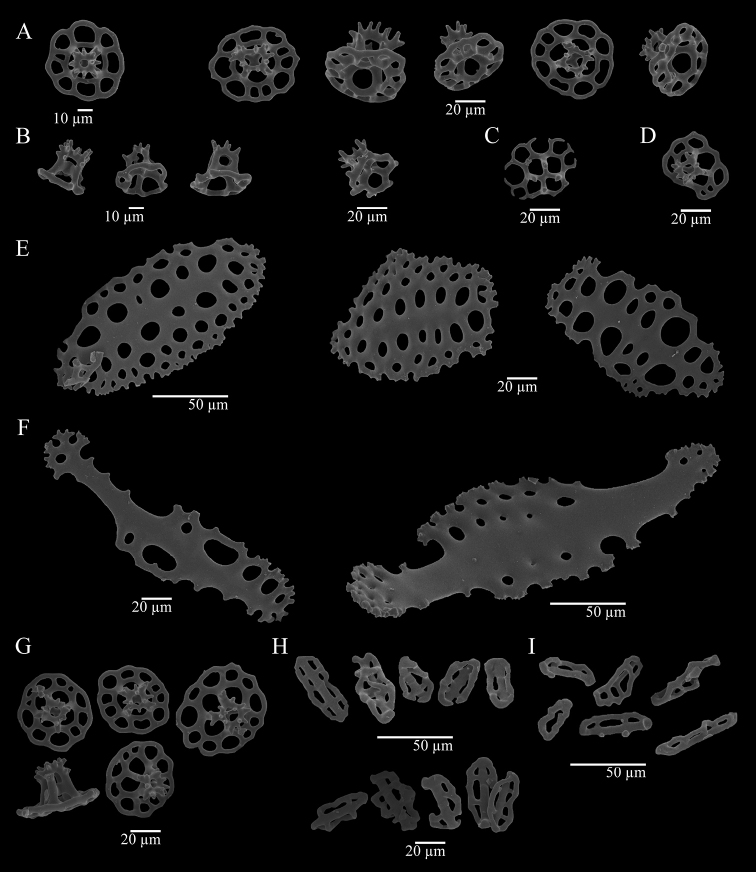
Holothuria (Lessonothuria) coronata sp. nov. Holotype, ICML-UNAM 18432. Ossicles of ventral tube feet (**A–F**) **A** tables **B** tables with reduced disc **C** table with reduced spire **D** ‘rare table’ **E** supporting rods **F** plates. Ossicles of ventral body wall (**G–I**) **G** tables **H** buttons **I** pseudo-buttons.

***Ossicles*.** Dorsal papillae with three types of tables, supporting rods, and a reduced terminal plate. The first type of table possesses circular disc (Fig. 2A), sometimes irregular (Fig. 2B), diameter of 50–70 µm, the rim is either smooth or spinous and turned up; four big central holes and 6–13 peripheral holes, sometimes a second series of smaller peripheral holes is present. Spire is low to medium and consists of four pillars, spiny crown with a central hole; single cross-beam. The second type of table (Fig. 2C) has a reduced disc with irregular rim, four central holes, and usually lacking peripheral holes, but some tables bear up to three peripheral holes. Spire is medium size (35–45 µm) and consists of four pillars, spiny crown with a central hole; single cross-beam. The third type of table (Fig. 2D) has reduced disc and smooth rim, four big central holes. Zero to seven peripheral holes, spire of four pillars; spiny crown. Curved supporting rods (140–240 µm) (Fig. 2E, F), the ends and the central part are widened and perforated; spiny edge. Reduced terminal plate, 50–60 µm wide. Anal papillae with three types of tables and supporting rods, these four types of ossicles are identical to those present in the dorsal papillae. Dorsal body wall with tables, buttons, and pseudo-buttons. Tables with circular disc (45–60 µm wide) (Fig. 2G), irregular rim, spinous or smooth; four central holes and eight to eleven peripheral holes, some tables present a second series of smaller peripheral holes. Spire low to medium sized consisting of four pillars, spiny crown with one central hole; single cross-beam. Some tables with button-shape and four big holes. Sometimes up to three peripheral holes are present, spire of four pillars, spiny crown. Irregular buttons (35–70 µm long) (Fig. 2H) with smooth edge, three to four pairs of holes. Pseudo-buttons (25–40 µm long) (Fig. 2I) with smooth and irregular edge, two to five holes. Ventral tube feet with three types of tables, plates, supporting rods, and terminal plate. The first type of table with circular disc (Fig. 3A), sometimes irregular (35–55 µm wide), and its rim is also irregular, whether spinose or smooth; four central holes and six to twelve peripheral holes. Spire low to medium with four pillars, spiny crown with central hole; single cross-beam. The second type of table bearing a reduced disc (30–40 µm wide) (Fig. 3B), four central holes and usually lacking peripheral holes, but rarely up to three peripheral holes are present. Medium size spire consisting of four pillars, spiny crown with central hole; single cross-beam. Tables rarely with a reduced spire (Fig. 3C). The third type are button-shaped tables (Fig. 3D) with spire of four pillars, spiny crown, disc with smooth rim and four big central holes. Perforated plates (Fig. 3E) with spiny irregular edge, 80–135 µm long. Curved supporting rods (130–230 µm) (Fig. 3F), the extremes and the central part are widened and perforated, irregular edge, sometimes with few spines. The terminal plates are bigger than the terminal plates from dorsal papillae. Ventral body wall with tables, buttons, and pseudo-buttons. Tables with circular disc (40–50 µm wide) (Fig. 3G) perforated by four central holes and eight to twelve peripheral holes. Spire low to medium with four pillars and spiny crown with central hole; single cross-beam. Button-shaped tables present, but scarcely. Disc with four big central holes and smooth rim, spire of four pillars, spiny crown. Buttons (40–50 µm length) (Fig. 3H) are irregular with three pairs of holes and smooth edge. Pseudo-buttons (Fig. 3I) with smooth edge and two to five holes (35–50 µm). Tentacles with slightly curved rods (Fig. 2J), both ends are spiny, without perforations (300–600 µm); some small rods are triradiated. Ossicles are not present in the longitudinal muscles, respiratory trees nor cloaca.

**Figure 4.**
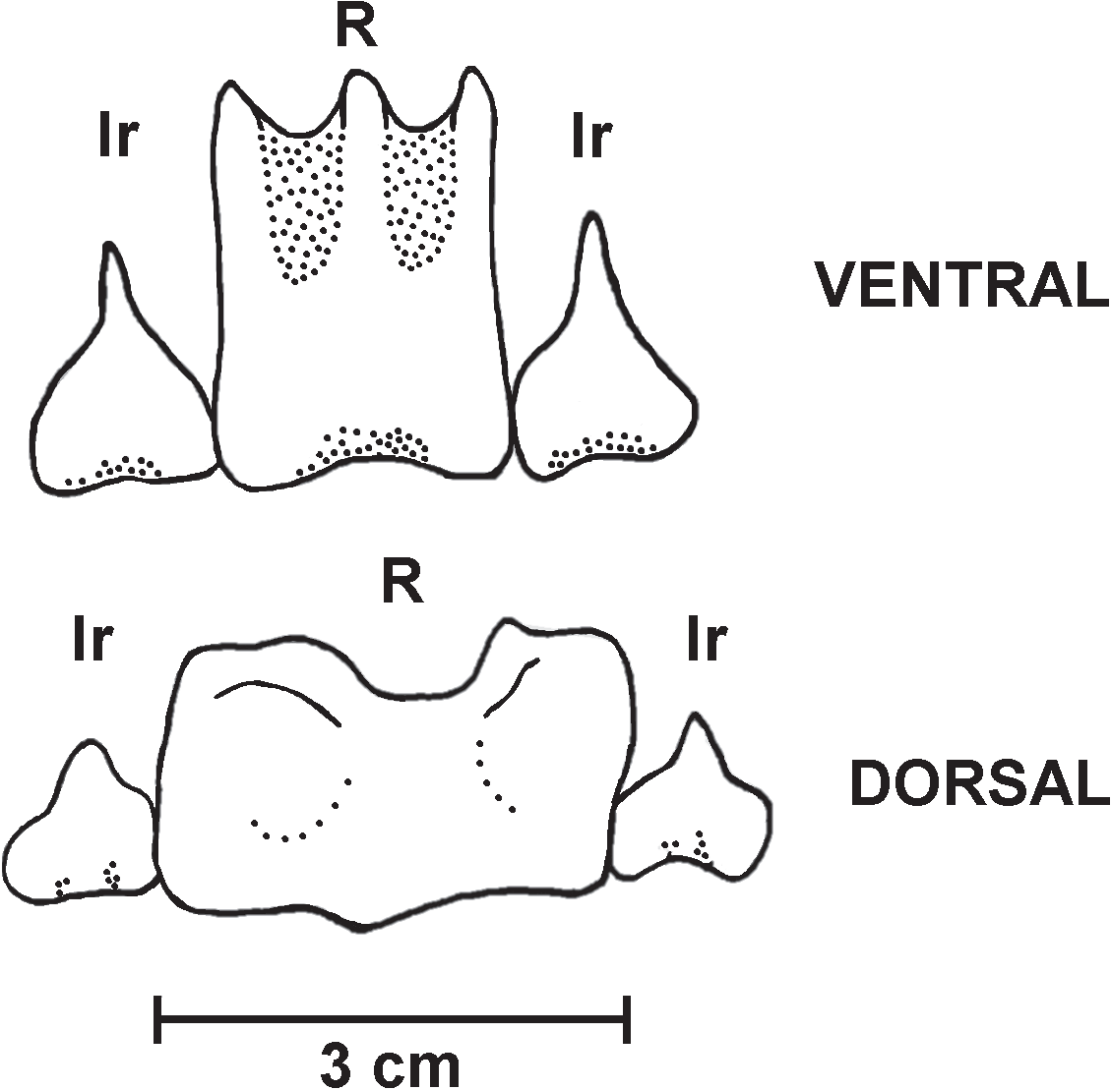
Holothuria (Lessonothuria) coronata sp. nov. Holotype, ICML-UNAM 18432. Calcareous ring. Single ventral and dorsal radials (R) and adjoining interradial plates (Ir).

###### Etymology.

The specific name refers to the crown of the table spires at the dorsal papillae, dorsal and ventral body wall, and ventral tube feet; such crowns exhibit a high shape diversity.

###### Ecology.

Holothuria (L.) coronata sp. nov. occurs at 0.5–10 m depth, hidden under rocks in a well-aerated environment. It is a burrowing, deposit feeding holothurian.

###### Geographical distribution.

Only known from Isla Socorro, Revillagigedo Archipelago, Pacific Ocean.

## ?Discussion

The number of tentacles and the presence of tables and buttons are characteristic of the subgenus Lessonothuria Deichmann, 1958. Holothuria (L.) coronata sp. nov. was grouped within this subgenus because of the presence of ossicles such as pseudo-buttons and tables with a disc whose rim is turned up (Rowe 1969).

Holothuria (L.) coronata sp. nov. is closely related to H. (L.) glandifera. The disc of the tables is spiny or smooth in both species, and Cherbonnier (1955) reported the presence of rudimentary tables in H. (L.) glandifera; such tables are similar to the tables with a reduced disc present in H. (L.) coronata sp. nov. According to Cherbonnier (1955), the body wall of H. (L.) glandifera also presents ‘rare tables’ that are similar to buttons; this kind of table is present in the ventral and dorsal body wall and ventral tube feet of H. (L.) coronata sp. nov. In addition, both species present some knobbed buttons (Cherbonnier 1955). Nonetheless, Cherbonnier (1955) did not mention the presence of a second series of peripheral holes on the disc of the tables of H. (L.) glandifera, a character which is present in some tables of H. (L.) coronata sp. nov., H. (L.) cumulus, H. (L.) duoturricula and H. (L.) multipilula (Clark 1921; Cherbonnier 1955, 1988; Liao 1975). Unlike H. (L.) glandifera, the tables of H. (L.) coronata sp. nov. bear a single cross-beam (Clark 1921; Cherbonnier 1955). The buttons of H. (L.) coronata sp. nov. are more irregular than those present in H. (L.) glandifera. The supporting rods (90–230 µm long) of the ventral ambulacral feet of H. (L.) glandifera don’t seem to be curved, their ends are perforated, and their edge is smooth; however, H. (L.) coronata sp. nov. presents longer and more complex supporting rods (130–230 µm long), curved, with a spiny edge, the ends and the central part are widened and perforated. The ventral tube feet of H. (L.) coronata sp. nov. and H. (L.) glandifera are supported by plates, while the plates of the ventral tube feet of H. (L.) coronata sp. nov. are wider and their edge is spinier (Cherbonnier 1955).

Holothuria (L.) coronata sp. nov. is also closely related to H. (L.) pardalis, but the tube feet supporting rods in H. (L.) pardalis are simpler than those in H. (L.) coronata sp. nov. and the table discs in H. (L.) pardalis have a spiny rim and are reduced, in addition to the fact that the spire is poorly developed, while in H. (L.) coronata sp. nov. the disc is generally well developed and the peripheral holes of the first series are large, the rim is smooth or spiny, but not as spiny as that of H. (L.) pardalis Selenka, 1867.

One species, Holothuria (L.) pardalis, has been reported inhabiting the Mexican Pacific. It is widely distributed, having been reported inhabiting the Indian Ocean and Western Pacific Ocean (Deichmann 1958; Samyn et al. 2019; Ahmed et al. 2020). Cherbonnier (1951) reported the presence of H. (L.) pardalis in the Gulf of California, Mexico. Seven years later, Deichmann (1958) reported H. (L.) pardalis inhabiting Tenacatita, Jalisco, Mexico and concluded that this species is not a permanent element of the fauna of the Mexican Pacific. She reviewed several specimens from different localities of the Central Eastern Pacific and reported ossicle variation among those specimens: ‘in some individuals the inner layer of buttons almost entirely composed of regular six-holed buttons, in others all deformed, twisted, incomplete or with few knobs on the surface'. Unfurnately, Deichmann (1958) did not give the information about where the illustrated material was collected, leaving the previous explanation without support.

Cherbonnier (1951) described minute white dots on the body wall of H. (L.) pardalis formed by clusters of ossicles, this character is also present in H. (L.) coronata sp. nov. According to the keys and the descriptions of Samyn et al. (2019) and Ahmed et al. (2020), we conclude that the ossicle illustrations provided by Cherbonnier (1951) truly belong to *H.pardalis* Selenka, 1867, due to the presence of peripheral holes in the majority of tables, the rods of the tentacles lacking holes, and the rods of the tube feet being curved. However, after reviewing the specimens of the subgenus Lessonothuria deposited in Colección Nacional de Equinodermos ‘Dra. Ma. Elena Caso Muñoz’, ICML, UNAM, we conclude that the statements of Deichmann (1958) are correct: H. (L.) pardalis does not naturally occur in the Mexican Pacific. The specimen reported by Solís-Marín et al. (2009) does not belong to *H.pardalis* but rather, H. (Vaneyothuria) zacae Deichmann, 1937. In conclusion, H. (L.) coronata sp. nov. is the second species of subgenus Lessonothuria that is distributed in the Mexican Pacific.

Holothuria (L.) coronata sp. nov. is clearly distinguished from other species of the subgenus by the presence of tables with a circular disc and big peripheral holes, sometimes with a second series of peripheral ones, disc with a spiny or smooth rim and a spire crossed by a single cross-beam, dorsal papillae and ventral tube feet with curved supporting rods with spiny edge, tentacles with non-perforated rods, all these characters have been used to differentiate species of the subgenus by various authors (Massin and Tomascik 1996; Ahmed et al. 2020).

## Supplementary Material

XML Treatment for Subgenus Lessonothuria

XML Treatment for Holothuria (Lessonothuria) coronata
